# Comparative Metabolic Profiling and Biological Evaluation of Essential Oils from *Conocarpus* Species: Antidiabetic, Antioxidant, and Antimicrobial Potential

**DOI:** 10.3390/plants14030464

**Published:** 2025-02-05

**Authors:** Aya Salim, Alya Ali Arasteh, Ruqaiya Sahrish, Danya Labash, Ali A. El-Keblawy, Haidy A. Gad, Naglaa S. Ashmawy

**Affiliations:** 1 Department of Pharmaceutical Sciences, College of Pharmacy, Gulf Medical University, Ajman P.O. Box 4184, United Arab Emirates; 2023mdd01@mygmu.ac.ae (A.S.); 2021ph02@mygmu.ac.ae (A.A.A.); 2021ph12@mygmu.ac.ae (R.S.); 2021ph07@mygmu.ac.ae (D.L.); 2 Department of Applied Biology, University of Sharjah, Sharjah P.O. Box 27272, United Arab Emirates; akeblawy@sharjah.ac.ae; 3 Department of Pharmacognosy, Faculty of Pharmacy, Ain Shams University, Cairo 11566, Egypt; haidygad@pharma.asu.edu.eg; 4 Department of Pharmacognosy, King Salman International University, El Tor 8701301, Egypt

**Keywords:** biological assessment, chemical profiling, *Conocarpus*, heat map, principal component analysis

## Abstract

Essential oils (EOs) are a diverse source of bioactive compounds with remarkable therapeutic potential. Despite their significance, *Conocarpus* EOs have been largely underexplored. This study provides a novel comparison of the metabolic profiles and biological activities of EOs from *C. lancifolius*, *C. erectus* green, and *C. erectus* silver leaves cultivated in the United Arab Emirates (UAE), offering unique insights into their distinct bioactive properties and potential therapeutic applications. EOs were extracted via hydro-distillation, analyzed using gas chromatography–mass spectrometry (GC-MS), and subjected to chemometric analysis. Their antioxidant (2,2-diphenyl-1-picrylhydrazyl (DPPH) and ferric reducing ability of plasma (FRAP) assays), antidiabetic (α-amylase and α-glucosidase inhibition), acetylcholinesterase (AChE) inhibition and antimicrobial activities were assessed. A total of 92 metabolites were identified, with heptacosane and nonacosane as key species discriminants. *C. lancifolius* EO showed the strongest α-amylase (IC_50_ 8.75 ± 0.54 µg/mL) and α-glucosidase (IC_50_ 22.31 ± 0.92 µg/mL) inhibitory activities, while *C. erectus* silver demonstrated superior antioxidant capacity (IC_50_ 349.78 ± 8.26 µg/mL, DPPH assay). *C. lancifolius* EO exhibited the best antimicrobial activity, particularly against *Staphylococcus aureus* (MIC 625 µg/mL). *C. erectus* silver EO inhibited *E. coli* and *C. albicans* (MIC 625 µg/mL). In contrast, *C. erectus* EOs showed no activity against *Aspergillus niger*. These findings highlight the potential of *Conocarpus* EOs as antioxidants and for managing diabetes that may be utilized either in nutraceuticals, dietary supplements or even in pharmaceutical formulations. Moreover, owing to significant antimicrobial activities, the EOs may be added to medical disinfectants and several pharmaceutical products. However, further, in vivo validation and pharmaceutical exploration is still needed.

## 1. Introduction

Plant-derived natural products have been utilized for centuries, tracing back to traditional medicine practices in certain cultures which have been passed down through generations [1]. These natural products harbour a diverse array of bioactive compounds, including flavonoids, alkaloids, terpenoids, carotenoids, and polyphenols, which possess various biological activities [2,3,4,5,6]. These compounds display anti-inflammatory [7], antioxidant [8], analgesic [9], antidiabetic [10], antibacterial [11], antifungal [12] and anticancer [13] effects. They can also act synergistically, enhancing their therapeutic efficacy and broadening their range of biological activity, which further increases their appeal to researchers [14,15,16]. Over time, there has been a growing emphasis on drug discovery from natural sources, with a focus on identifying biologically active compounds from plants for the treatment of various diseases and disorders [17].

Essential oils (EOs) play a significant role in drug discovery due to their richness in bioactive compounds, leading to a crucial therapeutic potential [18,19]. Various studies have reported the importance of plant EOs as a valuable reservoir for discovering and developing new pharmaceutical agents [20]. Their chemical complexity continues to make them a subject of extensive research as they have been associated with a wide range of biological activities and applications in both medicinal and cosmetic fields [21]. With respect to plant-based therapeutics, EOs contribute approximately 70%, representing a remarkably high proportion compared to other plant-derived therapeutic agents [22].

*Conocarpus*, also known as the Damas tree in the Gulf region, belongs to the Combretaceae family [23]. The Combretaceae is a large family consisting of 20 genera and 600 species of herbs, trees, and shrubs mainly distributed in Asia and Africa [24]. This family belongs to the order Myrtales and is known as the most utilized medicinal plant family in the order [25]. The *Conocarpus* genus comprises only two species, *Conocarpus erectus* and *Conocarpuslancifolius* [26], characterized by the ability to resist heat and salt, as well as drought, enabling them to survive especially in regions with high levels of heat and humidity [27]. *C. erectus* is native to the mangrove forest of Florida in North America, while *C. lancifolius* is native to the coastal and river areas of Somalia and Yemen [28]. *Conocarpus* has been widely used by different countries in folk medicine [29]. *C. lancifolius* leaf extract has been traditionally used to treat anemia, conjugative inflammatory, catarrh, diarrhea, diabetes, fever, bleeding, skin ulcers, and *syphilis orchitis* [30,31,32,33]. *C. erectus* species have been used traditionally for anemia, catarrh, conjunctivitis, diabetes, diarrhea, and fever [34].

*Conocarpus erectus* and *C. lancifolius* exhibit distinct adaptations to their environments, making them valuable sources of bioactive compounds for pharmaceutical and ecological applications [35,36,37]. *C. erectus* produces metabolites rich in polyphenols, flavonoids, and hydrolysable tannins, providing strong antioxidant and antibacterial properties. Its EOs contain compounds such as ellagic acid and polymethoxylated flavonoids, known for their potent bioactivities [38]. In contrast, *C. lancifolius* produces unique metabolites like trimethoxyellagic acid derivatives, kaempferol, and β-sitosterol glucosides, which demonstrate antioxidant, anti-inflammatory, cytotoxic, and antidiabetic effects due to their high phenolic and flavonoid contents [36,39]. Comparative studies in the UAE highlight these differences, with *C. lancifolius* emphasizing fruit extracts for polyphenolic extraction and *C. erectus* focusing on roots and leaves. Both species yield key bioactive compounds, such as vanillic acid, rutin hydrate, and quercetin, under optimized extraction conditions, positioning *C. erectus* as a source of potent antioxidant and antibacterial agents and *C. lancifolius* as a candidate for broader metabolic diversity and medicinal applications [40].

Direct comparisons between the green (*Conocarpus erectus* var. *erectus*) and silver (*Conocarpus erectus* var. *sericeus*) varieties remain limited, yet such studies are crucial for understanding their ecological and pharmaceutical potential. The green variety excels in photosynthesis under less extreme conditions, leveraging its higher chlorophyll content to sustain robust growth and efficient photosynthetic performance [41,42]. In contrast, the silver variety thrives in harsher environments, utilizing reflective trichomes to minimize heat absorption and water loss while promoting increased production of stress-related metabolites, such as terpenoids and phenolics [43]. A detailed exploration of these varieties would provide valuable insights into their unique metabolic strategies, offering opportunities to optimize their applications in ecological restoration and pharmaceutical development.

Despite the medical potential of the plant, it is still mostly used for landscaping and stabilizing soils. There are also limited data available regarding the EOs of *Conocarpus* species, which remain largely unexplored. Consequently, this study aimed to investigate and compare, for the first time, the metabolic profiles and biological activities of EOs isolated from the leaves of *Conocarpus* species grown in the United Arab Emirates. The EOs of *C. lancifolius* and the green and silver varieties of *C. erectus* may exhibit distinct chemical profiles and biological activities due to differences in their metabolic adaptations to environmental conditions. These differences in metabolic composition may result in variations in pharmacological properties, highlighting their potential for diverse therapeutic applications.

Consequently, this study evaluates the antioxidant, antidiabetic, and antimicrobial activities of EOs to uncover their therapeutic potential and promote broader pharmaceutical applications. It highlights the significance of identifying bioactive compounds to address global health challenges like diabetes, microbial resistance, and oxidative stress while advocating for the sustainable use of underutilized plant resources.

## 2. Results

### 2.1. GC/MS Analysis of the Essential Oils

GC-MS analysis of the EOs from *Conocarpus* species enabled the identification of 92 phytochemical components, accounting for 93.57%, 92.65%, and 94.9% of the EOs from *C. erectus* green leaves (CEG), *C. erectus* silver leaves (CES), and *C. lancifolius* leaves (CL), respectively (Table 1). The major components identified in the EOs of all three species were nonacosane, heptacosane, phytol, and farnesyl acetone, with variations in their relative abundances among the species.

Nonacosane was the predominant compound in the EOs of CEG and CES, constituting 23.36% and 24.54%, respectively, whereas its abundance in CL oil was lower at 9.76%. Heptacosane was the main constituent in CL oil, accounting for 23.32%, but was present at lower levels in CEG and CES oils, with percentages of 9.41% and 10.93%, respectively. Farnesyl acetone concentrations were measured as 6.64%, 4.55%, and 5.51% in the EOs of CEG, CES, and CL, respectively.

Phytol levels differed among the samples, with the highest percentage in CEG (13.56%), followed by CL (7.29%), and the lowest level in CES (0.85%). Unique compounds were identified in CL oil, including neophytadiene (0.26%), benzoic acid hexyl ester (0.18%), 2-methylnonacosane (0.09%), octadecanol acetate (0.08%), eicosanoic acid methyl ester (0.08%), 15-methylnonacosane (0.08%), hexacosanoic acid methyl ester (0.06%), heneicosanoic acid methyl ester (0.06%), and undecylcyclopentane (0.05%).

Distinctive compounds were also detected in the EOs of CEG and CES. Lupeol acetate (2.33%) and methyl tetracosanoate (0.12%) were present in CEG oil, whereas β-damascenone (0.16%), hexahydropseudoionone (0.11%), and palmitelaidic acid (0.10%) were specific to CES oil. These findings highlight the chemical diversity among the EOs of the three *Conocarpus* species.

### 2.2. Chemometric Analysis of the Essential Oils

**Figure 1:**
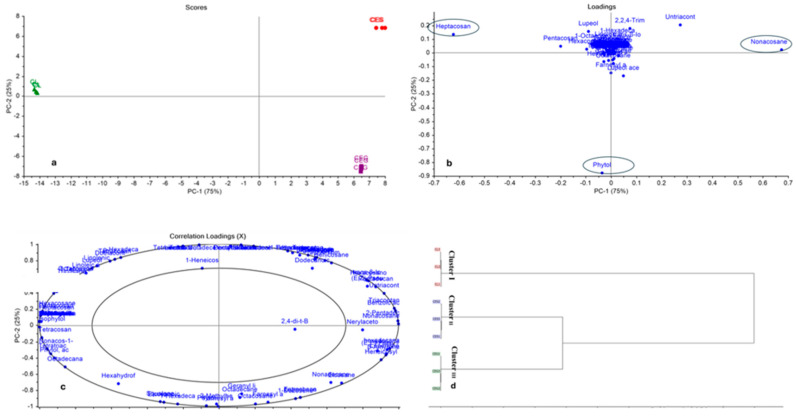
PCA score plot (**a**), loading plot (**b**), correlation loading plot (**c**), HCA (**d**) based on GC-MS identification of the chemical compositions of the EOs of CEG (C. *erectus* green), CES (*C. erectus* silver), and CL (*C. lancifolius*) leaves.

Both PCA and HCA were applied to allow for better discrimination of the different studied samples (CEG, CES, and CL). The PCA score and loading plots are shown in Figure 1a and Figure 1b, respectively. The PCA model explained 100% of the data variance, where PC1 accounted for 75% and PC2 explained 25% of the data variability. Complete segregation between the different studied samples was observed in the PCA score plot as each sample was located on a separate quadrant. CES was located on the upper right quadrant completely far away from the other samples, while CEG was positioned on the lower right quadrant, both on the positive side of PC1. However, the CL sample was allocated solely on the negative side of PC1, on the upper left quadrant. This confirmed the complete discrepancy in the metabolic profile between *C. lancifolius* and *C. erectus* green and silver leaves.

By investigation of the loading plot, Figure 1b, it was clarified that heptacosane was the key marker responsible for the segregation of *C. lancifolius* leaves (CL). However, nonacosane was the main metabolite with the highest impact in the separation of the *C. erectus* silver leaves (CES) sample. Although nonacosane was present at the same percentage in both *C. erectus* green leaves (CEG), and silver leaves (CES), it was able to discriminate only *C. erectus* silver leaves (CES) from green leaves (CEG). Conversely, it was observed that phytol was identified in lower amounts, which was the key marker that accounted for the separation of CEG. This finding was confirmed by the correlation loading plot shown in Figure 1c, which showed that all the identified compounds were computed for the displayed principal components, as the outer ellipse indicates 100% of the explained variance. Consequently, the whole metabolic profile, not solely the compounds identified in high percentages, is crucial for the segregation between closely related samples that exhibited similar qualitative and quantitative metabolic profiles.

**Figure 2:**
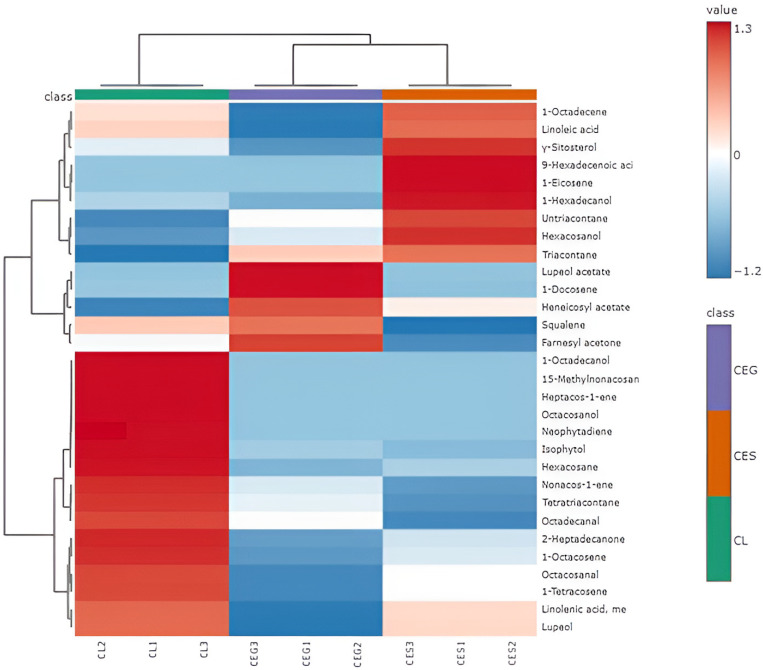
Heat map showing the differences in oil composition between the three *Conocarpous* species. CL (*C. lancifolius*), CEG (*C. erectus* green leaves), and CES (*C. erectus* silver leaves).

Additionally, HCA was also employed to verify the pattern obtained by PCA. The dendrograms displayed in Figure 1d revealed the same clustering relation as that of the PCA. CEG and CES were grouped in Cluster II, and III, respectively, closely related to each other. Conversely, Cluster I showed CL samples. A heat map (see Figure 2) was employed to demonstrate the distribution of all the data and to portray the comparative intensities of the various metabolites throughout the different samples.

### 2.3. Antioxidant Activity

In the present study, the antioxidant activity of the EOs derived from three *Conocarpus* plants CEG, CES, and CL was evaluated and reported for the first time using two complementary assays, namely, DPPH and FRAP, as summarized in Table 2.

The IC_50_ values of the EOs were compared to the standard ascorbic acid, which showed an IC_50_ of 10.20 ± 0.72 µg/mL in the DPPH assay and 20.88 ± 0.96 µg/mL in the FRAP assay. Among the three species, *C. erectus* silver leaves (CES) exhibited the strongest antioxidant activity, followed by *C. lancifolius* (CL), with *C. erectus* green leaves (CEG) showing the lowest activity. These findings are detailed in Table 2.

### 2.4. Enzyme Inhibitory Activities

**Figure 3:**
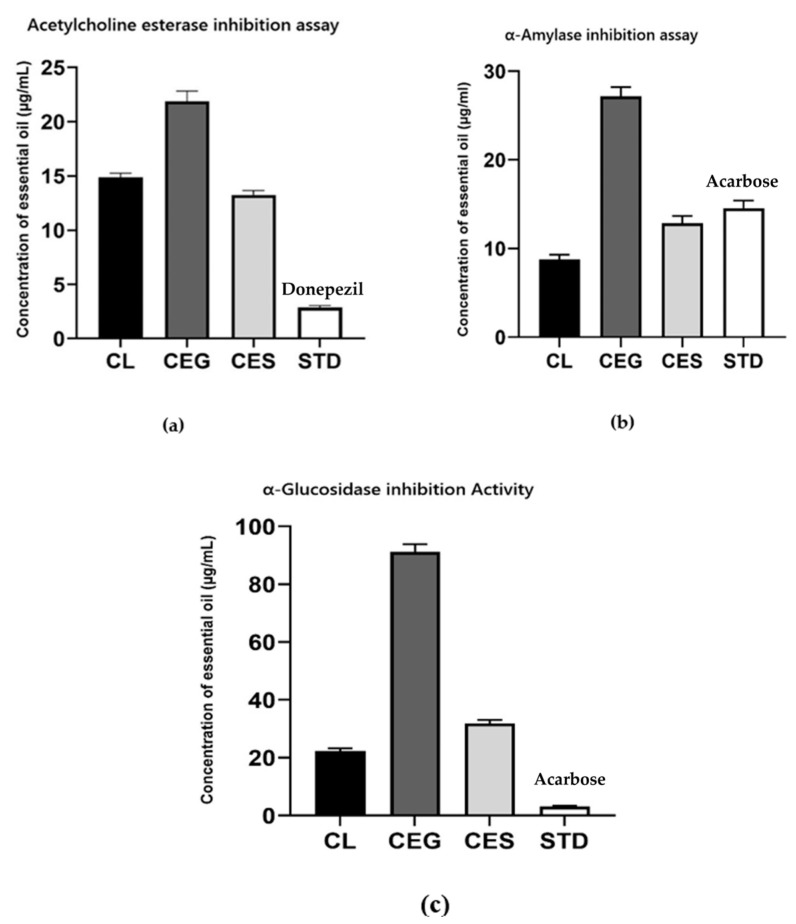
Enzyme inhibition activities of three *Conocarpus* sp. EOs: (**a**) ACE, (**b**) α-amylase, and (**c**) α-glucosidase. CL (*C. lancifolius*), CEG (*C. erectus* green leaves), and CES (*C. erectus* silver leaves).

The therapeutic efficacy of the EOs from the *Conocarpus* species was investigated for their potential in Alzheimer’s disease management and diabetes. This was achieved through the analysis of acetylcholinesterase (AChE), α-glucosidase, and α-amylase inhibition assays. The AChE enzyme is linked to AD development [44], while α-glucosidase and α-amylase are critical targets in diabetes management [45]. The enzyme inhibition activities of the EOs isolated from *Conocarpus* species are presented in Figure 3.

The EO from *C. lancifolius* exhibited the strongest α-amylase inhibition among the three species, with an IC_50_ value of 8.75 ± 0.54 µg/mL compared to the *C. erectus* green EOs (27.17 ± 1.05 µg/mL) and *C. erectus* silver EOs (12.83 ±0.83 µg/mL). Remarkably, *C. lancifolius* EOs also showed a lower IC_50_ value than the tested standard Acarbose (14.54 ± 0.86 µg/mL), underscoring its significant antidiabetic potential. Furthermore, *C. lancifolius* EOs also demonstrated superior α-glucosidase inhibition, with an IC_50_ value of 22.31 ± 0.92 µg/mL, compared to *C. erectus* green (91.09 ± 2.73 µg/mL) and C. erectus silver (31.79 ± 1.24 µg/mL) EOs. Regarding the acetylcholinesterase (AChE) inhibition activity of the tested EOs, the samples showed activity towards the AChE enzyme, with an IC_50_ value of 13.25 ± 0.41 µg/mL, 14.87 ± 0.38 µg/mL and 21.90 ± 0.93 µg/mL for CES, CEG, and CL, respectively, compared to the standard drug Donepezil (2.87± 0.19 µg/mL).

### 2.5. Antimicrobial Effect of Essential Oils

The results of the antimicrobial efficacy of EOs from the three *Conocarpus* species, summarized in Table 3 and Table 4, reveal that *C. lancifolius* EO exhibited the highest susceptibility (15 mm) against the Gram-positive bacteria *Staphylococcus aureus*. Among the *C. erectus* species, the silver variant showed the highest activity (15 mm) against the fungal strain *Candida albicans*, followed by the green variant (12 mm). However, the positive controls, Gentamycin for *Staphylococcus aureus* (24 mm) and Ketoconazole for *Candida albicans* (20 mm), demonstrated superior antimicrobial activity compared to the EOs.

The lowest susceptibility was observed with *C. lancifolius* EO against *Aspergillus niger* (9 mm), *C. erectus* silver EO against the Gram-negative bacteria *Bacillus subtilis* (9 mm), and *C. erectus* green EO against both *Staphylococcus aureus* and *E. coli* (10 mm). Notably, both *C. erectus* species displayed no activity against *Aspergillus niger*, and *C. erectus* green EO additionally showed no activity against the Gram-negative bacteria *Proteus vulgaris*.

Also, the findings presented in Table 4 indicate that the lowest concentration required to inhibit the growth of Gram-positive bacteria, specifically, *Staphylococcus aureus*, was 625 µg/mL, observed with the EO from *C. lancifolius*. Similarly, the lowest concentration needed to inhibit the growth of the Gram-negative bacteria *E. coli* was also 625 µg/mL, demonstrated by *C. erectus* silver EO. Furthermore, *C. erectus* silver EO displayed the lowest concentration for inhibiting fungal growth, specifically, *C. albicans*, at 625 µg/mL. However, *C. erectus* species showed no activity against *Aspergillus niger*, highlighting a limitation in their antifungal efficacy. In contrast, *C. lancifolius* EO exhibited activity against all the tested microorganisms.

## 3. Discussion

The GC-MS analysis of the EOs from *Conocarpus* species revealed significant variability in their phytochemical composition, reflecting species-specific differences and potential ecological adaptations. The identification of 92 components, accounting for over 90% of the total composition in all three EOs, underscores the chemical richness of these species. Such chemical diversity in essential oils has been documented in various plant species, highlighting the complex nature of these natural products [46]. Among the identified compounds, nonacosane and heptacosane were predominant, with their relative abundances varying among the species. Nonacosane was most abundant in the EOs of *C. erectus* green leaves (CEG) and silver leaves (CES), comprising 23.36% and 24.54%, respectively, but was present at significantly lower levels in *C. lancifolius* (CL) oil (9.76%). Conversely, heptacosane dominated the CL oil (23.32%) but was less prominent in CEG and CES oils. These findings suggest species-specific biosynthetic pathways, possibly influenced by genetic and ecological factors [47].

Phytol, a diterpene alcohol, exhibited significant variability, being highly abundant in CEG (13.56%), moderately present in CL (7.29%), and nearly absent in CES (0.85%). This pattern suggests that phytol may play distinct roles in the ecological or physiological functions of these species. Variability in terpenoid compounds within essential oils has been associated with environmental factors and plant developmental stages [48,49].

Unique compounds were also identified in each species, highlighting the chemical diversity among the oils. For instance, neophytadiene, benzoic acid hexyl ester, and 2-methylnonacosane were exclusive to CL oil. Similarly, lupeol acetate and methyl tetracosanoate were present only in CEG oil, while β-damascenone, hexahydropseudoionone, and palmitelaidic acid were detected solely in CES oil. These distinctive profiles may contribute to the specific ecological interactions and potential pharmacological properties of each species [50].

The role of the broader metabolic profile in distinguishing closely related species was further supported by multivariate statistical analysis, as shown in Figure 1. PCA revealed that nonacosane and heptacosane were critical markers for the segregation of CES and CL, respectively, while phytol was the key compound separating CEG. This finding aligns with studies emphasizing the importance of comprehensive metabolic profiling in differentiating species with overlapping phytochemical constituents [51]. The segregation patterns observed in the PCA loading plot suggest that the metabolic variability is not solely governed by the presence of dominant compounds but also by the contributions of minor metabolites. For example, the discrimination of CEG by phytol, despite its lower concentration, underscores the role of specific metabolites as unique chemical signatures for certain plant tissues. This highlights the interplay between primary and secondary metabolites in defining the metabolic fingerprint of each species, which can be influenced by environmental conditions, genetic factors, and evolutionary adaptations [52]. Furthermore, the ability of nonacosane to differentiate CES, despite being present in both CEG and CES samples, highlights the complexity of metabolic interactions and the importance of multivariate tools in untangling these relationships. Such findings are significant not only for understanding the ecological and physiological roles of these compounds in the plant’s survival strategies but also for their potential applications in chemotaxonomy, pharmacology, and industry. The identification of these markers could serve as a foundation for further studies into the biosynthetic pathways that generate such variability and how they may be leveraged for targeted applications, such as the development of bioactive compounds or the improvement of stress tolerance in plants. Ultimately, these results confirmed that chemometric analysis is essential to visualize and interpret complex multidimensional data [53].

Moreover, in this study, the antioxidant, enzyme inhibition, and antimicrobial activities of the EOs from the three species were investigated. The antioxidant activities of plant extracts from the *Conocarpus* genus have been reported previously. The defatted methanol extracts of *C. erectus* from various plant parts demonstrated significant free radical scavenging activity against DPPH radicals, with SC_50_ values ranging from 6.47 to 9.4 µg/mL [54]. Additionally, the methanol extract of the dried aerial parts of *C. lancifolius* exhibited DPPH and hydroxyl radical scavenging activities of 93.35% [23].

*C. erectus* silver (CES) leaves EO showed a superior antioxidant effect, which may be attributed to the presence of some compounds uniquely in its EO profile, like β-damascenone, which has been reported for its antioxidant capacity, being able to activate Nrf2 and inhibit NF-κB acting as an electrophilic compound [55]. Moreover, hexahydropseudoionone is another compound that is suggested to be responsible for the superiority of the CES antioxidant effect because of its isoprene structure [56]. These results were in accordance with various literature that reported the wide utilization of plant EOs for their antioxidant effects [57], which are attributed to their rich content of bioactive compounds [58]. These natural antioxidants play a crucial role in scavenging free radicals and mitigating oxidative stress associated with various chronic diseases and cellular damage [59].

Regarding enzyme inhibition activities, EO from *C. lancifolius* exhibited the strongest α-amylase inhibition among the three species. Moreover, *C. lancifolius* EO also exhibited the best α-glucosidase inhibition among the three EOs (Figure 3b,c). This superior activity of CL essential oil may be attributed to its high concentrations of heptacosane (23.32%) and nonacosane (23.36%), as hydrocarbons-rich EOs have been reported to possess notable antidiabetic properties [60,61].

Moreover, all three tested EOs showed inhibitory activities towards the acetylcholinesterase enzyme (Figure 3a), with CES exhibiting the strongest inhibition, suggesting variability in enzyme interaction among the samples. These results indicate that the oil samples possess neuroprotective potential, making them promising candidates for managing neurodegenerative disorders. This inhibitory activity could be attributed to the unique hydrocarbon composition of bioactive compounds, which warrants further in vivo investigation.

Additionally, the antimicrobial activity of the EOs from the three *Conocarpus* species was tested in this study and the EOs demonstrated varying levels of efficacy against the tested microbial strains. *C. lancifolius* EO exhibited better susceptibility against all tested microorganisms, with higher efficacy against Gram-positive *Staphylococcus aureus* (Table 4). Among the *C. erectus* species, the silver variant displayed superior antifungal activity against *Candida albicans* (Table 4), while the green variant exhibited limited activity across most strains. Both *C. erectus* species lacked activity against *Aspergillus niger*, underscoring a limitation in their antifungal spectrum.

The higher susceptibility of EOs against Gram-positive bacteria compared to Gram-negative bacteria can be attributed to differences in cell wall structure. Gram-positive bacteria have a thick peptidoglycan layer that is more accessible to antibacterial agents, whereas the outer membrane of Gram-negative bacteria, composed of lipopolysaccharides, acts as an additional barrier, limiting the penetration of antimicrobial compounds [62]. This structural disparity likely explains the observed variation in susceptibility across microbial strains.

Despite these findings, the MIC values of the EOs (Table 4) were not significant when compared to the controls. This highlights the need for further investigation into the chemical composition and potential synergistic effects within these EOs to better understand their antimicrobial properties.

## 4. Methods

### 4.1. Plant Collection

Three plants of the *Conocarpus* genus, namely, *C. lancifolius* and *C. erectus* green leaves and *C. erectus* silver leaves, were collected in May 2024 from their natural habitats in the desert region of the United Arab Emirates, ensuring representative sampling across different ecological niches. The plants were botanically identified by Prof. Ali Al-Qabalawi, Professor of Botany at the University of Sharjah. The specimens were identified, tagged, and documented according to standard botanical procedures. Voucher specimens were deposited in the College of Pharmacy, Gulf Medical University, UAE. The symbols of CL, CEG, and CES are assigned to *C. lancifolius*, *C. erectus* green leaves, and *C. erectus* silver leaves, respectively.

### 4.2. Plant Drying

Collected plant materials were cleaned to remove extraneous matter and then leaves were separated and air dried under controlled conditions to preserve their chemical integrity.

### 4.3. Essential Oils Isolation

A total of 500 g of leaves from each of the three *Conocarpus* plants was subjected to hydro-distillation for 4 h using a Clevenger-type apparatus. The EOs obtained were collected, desiccated to remove moisture, and stored in sealed vials at −4 °C, protected from light, to preserve their integrity for further analysis. The experiment was performed in triplicate to ensure reproducibility.

### 4.4. GC/MS Analysis of Essential Oils

The EOs isolated from the three *Conocarpus* plants were analyzed via gas chromatography-mass spectrometry (GC/MS) using Shimadzu GC/MS-QP 2010 (Kyoto, Japan) coupled to a mass spectrometer (SSQ 7000 quadrupole: Thermo-Finnigan, Bremen, Germany) for metabolic profiling. The analysis was performed using a capillary column with a 0.25 mm internal diameter and a 0.25 µm film thickness. The GC temperature program was set as follows: an initial temperature of 45 °C was maintained for 2 min, followed by an increase at a rate of 5 °C/min to 300 °C, which was held for 5 min. The injector temperature was set at 250 °C, and the detector temperature at 280 °C. EOs were diluted to a concentration of 1% (*v*/*v*) in n-hexane, and 1 µL of the prepared sample was injected automatically with a 1:15 split ratio. Helium was used as the carrier gas at a flow rate of 1.41 mL/min. The mass spectrometer operated with an ionization voltage of 70 eV, an ion source temperature of 200 °C, and a scan range of 35–500 *m*/*z* [63].

### 4.5. Identification of the Oil Components

The components of the EOs were identified by comparing their GC/MS spectra, fragmentation patterns, and retention indices with previously reported data in the literature [64]. Retention indices were calculated relative to a homologous series of n-alkanes (C8–C28) analyzed under identical conditions. The resulting data were cross-referenced with the NIST-11 and Wiley Registry of Mass Spectral Databases, as well as relevant literature, to ensure accurate characterization [65].

### 4.6. Chemometric Analysis

GC-MS metabolic profiles of the three EOs were investigated by chemometric analysis, where principal component analysis (PCA) was performed as the prime step in data analysis to investigate the discrepancies and resemblances between *Conocarpus erectus* green leaves (CEG), *Conocarpus erectus* silver leaves (CES), and *Conocarpus lancifolius* leaves (CL), and to distinguish the crucial metabolites responsible for the pattern obtained. Hierarchal cluster analysis (HCA) was then applied to explore the clustering patterns operated by the complete linkage way. This demonstration is more efficient when the distance between samples is handled by the Euclidean method. A matrix of the nine samples (3 samples X 3 replicas) multiplied by 93 variables (GC/MS peak area %) was built in MS Excel^®^ (Microsoft 365) and then subjected to chemometric analysis. PCA and HCA were performed by CAMO’s Unscrambler^®^ X 10.4 software (Computer-Aided Modeling, AS, Oslo, Norway). The heat map was generated using MetaboAnalyst 6.0 (https://www.metaboanalyst.ca/) [66].

### 4.7. Antioxidant Assay

The antioxidant potential of the EOs was evaluated using the 2,2-diphenyl-1-picrylhydrazyl (DPPH) free radical scavenging assay and the ferric reducing antioxidant power (FRAP) assay. Both assays were conducted in triplicate, and the average values were calculated for analysis.

#### 4.7.1. DPPH Radical Scavenging Activity

A methanol solution of (DPPH) radical was freshly prepared (0.004% *w*/*v*) and then stored in the dark at 10 °C. A methanol solution of a test compound was also prepared. A 40 µL aliquot from the methanol solution was added to 3 mL of DPPH solution. Absorbance measurements were recorded instantly with a UV-visible spectrophotometer (Milton Roy, Spectronic 1201, Ivyland, PA, USA). The decrease in absorbance was determined continuously at 515 nm, and the data were recorded at 1 min intervals until the absorbance stabilized (16 min). The control was determined as the absorbance of the DPPH radical without antioxidants. The reference compound used for comparison was ascorbic acid, and its absorbance was also measured under identical conditions. The reducing capability of the EOs samples was compared to that of ascorbic acid to evaluate their antioxidant potential. The measurements were performed in triplicate, and the average values were calculated. The measurements were performed in three replicates and then the average was taken. The percentage inhibition (PI) of the DPPH radical was calculated using the following formula [57]:

PI = [(AC − AT)/AC × 100](1)

AC = Absorbance of the control at t = 0 min; AT = absorbance of the sample + DPPH at t = 16 min.

The half-maximal inhibitory concentration (IC_50_) and the concentration for 50% DPPH radical scavenging activity were determined from dose–response curves using GraphPad Prism software (version 7, San Diego, CA, USA).

#### 4.7.2. Ferric Reducing Antioxidant Power (FRAP) Assay

The potential antioxidant activity of the EOs was assessed based on its capacity to reduce ferric ions (Fe^3^⁺) to ferrous ions (Fe^2^⁺). The reducing power of the EOs was determined using a method involving the reduction of ferricyanide in the presence of the essential oil sample [60]. The essential oil sample (1 mg/mL) dissolved in 1 mL of methanol was mixed with 2.5 mL of 0.2 M sodium phosphate buffer (pH 6.6) and 2.5 mL of potassium ferricyanide [K_3_Fe(CN)_6_] (1%, *w*/*v*). The mixture was then incubated at 50 °C for 20 min. Afterward, the reaction mixture was acidified with 2.5 mL of trichloroacetic acid (10%, *w*/*v*) and centrifuged at 1000× *g* for 10 min. From the resulting supernatant, 2.5 mL was collected and mixed with 2.5 mL of deionized water and 0.5 mL of freshly prepared ferric chloride solution (0.1%, *w*/*v*). The absorbance of the final solution was measured at 700 nm using a spectrophotometer (Milton Roy, Houston, TX, USA, Spectronic 1201) against a blank. The percentage of reducing capability was calculated using the following equation [57]:

Reducing capability (%) = 100 − [(A_o_ − A_s_)/Ao × 100] (2)

A_0_: absorbance of the control solution. A_s_: sample absorbance.

The half-maximal inhibitory concentration (IC_50_) and the concentration needed to have 50% radical scavenging activity were determined from graphic plots of the dose–response curve using GraphPad Prism software (San Diego, CA, USA).

### 4.8. Enzyme Inhibition Assays

The enzyme inhibition activities of the three essential oil samples were assessed against three enzymes, namely, α-amylase, α-glucosidase, and acetylcholine esterase.

#### 4.8.1. α-Amylase Inhibition Assay

The α-amylase inhibition activity of the EOs was tested as per the 3,5-dinitrosalicylic acid (DNSA) method [61]. The tested samples were first dissolved in 10% DMSO and then dissolved in buffer Na_2_HPO_4_/NaH_2_ PO_4_ (0.02 M), NaCl (0.006 M) at pH 6.9, resulting in concentrations that ranged from 10 to 1000 μg/mL. Then, 200 μL of α-amylase solution (2 units/mL) was mixed with 200 μL of the test sample and incubated for 10 min at 30 °C. Afterward, 200 μL of the starch solution (1% in water (*w*/*v*)) was added to each tube and then incubated for 3 min. To stop the reaction, 200 μL of DNSA reagent (12 g of sodium potassium tartrate tetrahydrate in 8.0 mL of 2 M NaOH and 20 mL of 96 mM of 3,5-dinitrosalicylic acid solution) was added. After stopping the reaction, the mixture was then inserted in a water bath at 85–90 °C for 10 min to boil. The mixture was left to cool down to room temperature and then diluted using 5 mL of distilled water. The absorbance was measured using a UV-Visible spectrophotometer at 540 nm. The blank used in the assay was buffer only, and the positive control was Acarbose, tested at concentrations ranging from 100 μg/mL to 2 μg/mL. The α-amylase inhibitory activity was calculated using the equation given below:

% α-amylase inhibition = 100 [(Abs 100% control − Abs Sample)/Abs100% Control)](3)

The percentage of α-amylase inhibition was then plotted against the concentration of the tested essential oil and the IC_50_ values were obtained from the graph [40].

#### 4.8.2. α-Glucosidase Inhibition Assay

The α-glucosidase inhibitory activity of the EOs was assessed by preparing 50 μL of α-glucosidase enzyme solution (1 U/mL) in 0.1 M phosphate buffer (pH 6.9), mixed with 250 μL of the same buffer to achieve a concentration range of 0.5 to 1000 μg/mL. Subsequently, 100 μL of the test sample, prepared in a concentration range of 2 to 4000 μg/mL, was added to the enzyme–buffer mixture. The reaction mixture was pre-incubated at 37 °C for 20 min. Following pre-incubation, 10 μL of 10 mM p-nitrophenyl-α-D-glucopyranoside (pNPG) solution, prepared in 0.1 M phosphate buffer (pH 6.9), was added, and the mixture was incubated again at 37 °C for an additional 30 min. To terminate the reaction, 650 μL of 1 M sodium carbonate was added. The absorbance of the final solution was measured at 405 nm using a spectrophotometer (Amersham Biosciences, Piscataway, NJ, USA) [40].

The percentage of α-glucosidase inhibition was calculated using the following formula:

% α-glucosidase Inhibition = [(A_405_Control − A_405_Treatment)/A_405_Control] × 100 (4)

where A_405_ Control: absorbance at 405 nm for control (buffer only)**;** A_405_ Treatment: absorbance at 405 nm for the tested sample.

#### 4.8.3. Acetylcholinesterase Inhibition Assay

The acetylcholinesterase (AChE) inhibitory activity was evaluated using a modified 96-well microplate assay based on Ellman’s method [62], as further refined by Rhee et al. (2001) [63]. This sensitive method measures the production of thiocholine from the hydrolysis of acetylthiocholine, which reacts with 5,5-dithiobis (2-nitrobenzoic acid) (DTNB or Ellman’s reagent) to produce a yellow-colored product detectable at 405 nm. The assay was performed using three buffer solutions: 50 mM Tris-HCl buffer at pH 8.0 (Solution A), the same buffer containing 0.1% bovine serum albumin (Solution B), and Tris-HCl buffer containing 0.1 M NaCl and 0.02 M MgCl_2_·6H_2_O (Solution C).

In each well of a 96-well microplate, 25 µL of acetylthiocholine iodide (15 µM), 125 µL of DTNB (3 µM) of Solution C, 50 µL of Solution B, and 25 µL of the test compound dissolved in methanol and diluted with Solution A (at concentrations ranging from 0.5 to 1000 µg/mL) were added. The absorbance was measured at 405 nm for 30 s using a Microplate TS-800 reader (BioTek, Winooski, VT, USA). Following this, 25 µL of AChE solution (0.22 U/mL) was added to each well, and the absorbance was recorded every 5 min for a total of four readings during incubation. The percentage inhibition of AChE activity was calculated by comparing the reaction rates of the test samples to the negative control (10% methanol in Solution A, which represented 100% enzyme activity). Donepezil was used as a positive control at concentrations ranging from 0.5 to 1000 µg/mL. All experiments were conducted in triplicate, and the results were expressed as mean ± standard deviation.

### 4.9. Antimicrobial Activity Assay

#### 4.9.1. Microorganisms

In this investigation, four bacterial strains were utilized. These strains included two Gram-positive bacteria, *Staphylococcus aureus* ATCC 25923 and *Bacillus subtilis* NRRL B-543, as well as two Gram-negative bacteria, *Escherichia coli* ATCC 25922 and Proteus vulgaris ATCC 13315. Additionally, the study included two fungal strains, one of which is a filamentous fungus: *Aspergillus niger*, and another yeast species: *Candida albicans* ATCC 10231.

#### 4.9.2. Disc-Diffusion Test

The antimicrobial activity of the plant EOs was tested against Gram-positive bacterial species (*Staphylococcus aureus* and *Bacillus subtilis*), Gram-negative bacterial species (*Escherichia coli*, *Proteus vulgaris*), as well as against fungi, including one filamentous fungus (*Aspergillus fumigatus*) and one yeast species (*Candida albicans*), by a modified well diffusion method. From each sample, 100 µL (at 10 mg/mL) was added to each well (6 mm diameter holes cut in the agar gel). The plates were then incubated for 24–48 h at 37 °C for bacteria and yeast, and for 48 h at 28 °C for filamentous fungi. Afterward, the microorganisms’ growth was noted. The diameters for the inhibition zone were measured in millimeters and served as an indication of antimicrobial activity. The size of this zone is proportional to antimicrobial activity. The negative control used was DMSO and it was tested with samples for any possible interference on the microbial growth, which showed no interference. The positive controls used in the experiment included gentamycin as a standard antibacterial drug and ketoconazole as a standard antifungal drug [67].

#### 4.9.3. Minimum Inhibitory Concentration Test

The minimum inhibitory concentration (MIC) determination was performed for the EOs using the microtitre broth dilution method. The serial dilution was performed according to Clinical and Laboratory Standards Institute (CLSI) guidelines. A stock solution was prepared by diluting the samples of EOs with dimethylsulphoxide (DMSO) to a final concentration of 20 mg/mL and kept in a 1.5 mL microcentrifuge tube. The stock solution was used to create a serial dilution with a range from 10,000 μg/mL to 0.61 μg/mL using Mueller–Hinton broth (Becton Dickinson, Sparks, MD, USA) in 96-well microplates. The bacterial suspension was created from a 24 h culture, and it contained around 5 × 10^5^ colony-forming units/mL. In the microplate, 100 µL of the bacterial suspension was added to each and two wells were kept for both sterility and growth controls. The microplates were then incubated for 24 h for the bacteria and 48 h for the filamentous fungi at 37 °C. After incubation, 40 μL of a 0.4 mg/mL solution of INT was added to each well, serving as an indicator of microbial growth. The plates were then incubated again at 37 °C for 30 min (bacteria) and 24 h (filamentous fungi). The lowest concentration from each sample exhibiting no microbial growth was determined as the minimum inhibitory concentration (MIC). The concentration which exhibited the first inhibition of microbial growth was taken as the MIC value. MIC values were determined in duplicates and the experiment was repeated as confirmation. The negative control used was DMSO only, and the positive controls were similar to the disc-diffusion method [68].

### 4.10. Statistical Analysis

All experiments were carried out three times in triplicate. Data are expressed as mean ± standard deviation. The IC_50_ values were calculated, and the results were presented using GraphPad Prism^®^ software (Version 7, graph-Pad software Inc., San Diego, CA, USA).

## 5. Conclusions

This study provides the first comparative analysis of the metabolic profiles and biological activities of EOs derived from the leaves of *Conocarpus* species cultivated in the UAE. The findings highlight significant chemical diversity among the EOs, which was confirmed by applying chemometric analysis, with *C. lancifolius* demonstrating superior α-amylase and α-glucosidase inhibition, underscoring its antidiabetic potential. Similarly, *C. erectus* silver showed remarkable antioxidant activity, which can be attributed to its unique phytochemical composition, including compounds such as β-damascenone and hexahydropseudoionone. While the antimicrobial effects were generally modest, *C. lancifolius* exhibited broader activity compared to the other species, reinforcing its potential as a source of bioactive compounds. Regarding antimicrobial activity, *C. lancifolius* EO inhibited all tested microorganisms, with the highest activity against *Staphylococcus aureus* (MIC of 625 µg/mL), while *C. erectus* silver EO demonstrated activity against both *E. coli* and *C. albicans*, with MIC values of 625 µg/mL. On the other hand, *C. erectus* EOs lacked activity against *Aspergillus niger*. These results highlight varying antimicrobial potentials among the species.

These results underline the therapeutic promise of *Conocarpus* EOs, particularly in managing diabetes and oxidative stress-related disorders. However, the lower antimicrobial potency relative to standard controls suggests a need for further exploration of synergistic effects and optimization of their use in pharmaceutical formulations. Future studies should also consider in vivo models and advanced bioassays to validate and expand the potential applications of these EOs.

## Figures and Tables

**Table 1:** Chemical composition of essential oils based on GC/MS analysis (n = 3).

	KI		Compound		Relative Abundance % ± SD		
No.	Cal.	Rep.		Molecular Formula	CEG	CES	CL
1.	1377	1374	Decanoic acid	C_12_H_20_O_2_	-	0.14 ± 0.05	0.08 ± 0.02
2.	1389	1388	β-Damascenone	C_13_H_18_O	-	0.16 ± 0.03	-
3.	1407	1408	Hexahydropseudoionone	C_13_H_26_O	-	0.11 ± 0.03	-
4.	1433	1432	α-Ionone	C_13_H_20_O	0.19 ± 0.07	0.16 ± 0.02	0.05 ± 0.08
5.	1458	1457	Nerylacetone	C_13_H_22_O	2.83 ± 0.04	2.74 ± 0.04	2.61 ± 0.14
6.	1494	1494	trans-β-Ionone	C_13_H_20_O	1.77 ± 0.09	2.88 ± 0.06	1.07 ± 0.04
7.	1502	1500	PentadecaneI8II	C_15_H_32_	-	0.15 ± 0.05	0.07 ± 0.01
8.	1515	1518	Tridecanal	C_13_H_26_O	-	0.16 ± 0.07	0.06 ± 0.01
9.	1521	1519	2,4-di-t-Butylphenol	C_14_H_22_O	0.47 ± 0.07	1.10 ± 0.02	0.17 ± 0.08
10.	1534	1535	(E,Z)-Pseudoionone	C_13_H_20_O	0.65 ± 0.04	0.79 ± 0.06	0.49 ± 0.05
11.	1574	1574	Dodecanoic acid	C_12_H_24_O_2_	0.46 ± 0.05	0.57 ± 0.09	0.45 ± 0.04
12.	1584	1581	Benzoic acid, hexyl ester	C_13_H_18_O_2_	-	-	0.18 ± 0.09
13.	1591	1590	(E,E)-Pseudoionone	C_13_H_20_O	0.68 ± 0.02	0.63 ± 0.05	0.53 ± 0.03
14.	1600	1588	2,2,4-Trimethyl-1,3-pentanediol ester	C_16_H_30_O_4_	0.63 ± 0.03	3.36 ± 0.07	0.42 ± 0.06
15.	1610	1613	Tetradecanal	C_14_H_28_O	-	0.29 ± 0.03	0.04 ± 0.03
16.	1659	1656	Undecylcyclopentane	C_16_H_32_	-	-	0.05 ± 0.03
17.	1665	1666	2-Methylhexadecane	C_17_H_36_	0.16 ± 0.02	0.09 ± 0.05	0.12 ± 0.03
18.	1674	1680	13-Methyltetradecanal	C_15_H_30_O	1.14 ± 0.07	0.95 ± 0.05	0.81 ± 0.08
19.	1698	1698	2-Pentadecanone	C_15_H_30_O	0.27 ± 0.04	0.29 ± 0.01	0.15 ± 0.06
20.	1702	1700	Heptadecane	C_17_H_36_	0.28 ± 0.08	0.31 ± 0.02	0.28 ± 0.01
21.	1709	1710	Pentadecanal	C_15_H_30_O	0.22 ± 0.04	0.31 ± 0.04	0.13 ± 0.04
22.	1750	1749	Farnesyl alcohol	C_15_H_26_O	1.00 ± 0.13	0.31 ± 0.05	0.54 ± 0.15
23.	1770	1771	Tetradecanoic acid	C_14_H_28_O_2_	-	0.60 ± 0.05	0.48 ± 0.01
24.	1791	1806	1-Octadecene	C_18_H_36_	0.59 ± 0.12	1.01 ± 0.05	0.86 ± 0.07
25.	1800	1800	Octadecane	C_18_H_38_	0.4 ± 0.08	0.31 ± 0.05	0.37 ± 0.04
26.	1805	1811	Hexadecanal	C_16_H_32_O	0.5 ± 0.11	0.43 ± 0.06	0.28 ± 0.03
27.	1841	1837	Neophytadiene	C_20_H_38_	-	-	0.26 ± 0.03
28.	1849	1848	Hexahydrofarnesyl acetone	C_18_H_36_O	3.28 ± 0.08	2.31 ± 0.03	3.83 ± 0.03
29.	1869	1860	Benzoic acid, 2-phenylethyl ester	C_15_H_14_O	0.27 ± 0.08	0.28 ± 0.02	0.24 ± 0.03
30.	1876	1876	1-Hexadecanol	C_16_H_34_O	0.67 ± 0.09	1.52 ± 0.04	0.29 ± 0.08
31.	1896	1900	1-Nonadecene	C_19_H_38_	-	0.81 ± 0.04	0.30 ± 0.06
32.	1902	1900	Nonadecane	C_19_H_40_	0.23 ± 0.05	0.15 ± 0.05	0.12 ± 0.03
33.	1907	1906	2-Heptadecanone	C_17_H_34_O	-	0.14 ± 0.03	0.04 ± 0.03
35.	1926	1927	Farnesyl acetone	C_18_H_30_O	6.64 ± 0.13	4.55 ± 0.12	5.51 ± 0.04
36.	1945	1942	Palmitelaidic acid	C_16_H_30_O_2_	-	0.10 ± 0.04	-
37.	1951	1950	Isophytol	C_20_H_40_O	0.25 ± 0.04	0.20 ± 0.08	0.82 ± 0.06
38.	1971	1971	n-Hexadecanoic acid	C_16_H_32_O_2_	2.89 ± 0.11	2.96 ± 0.07	2.97 ± 0.03
39.	1994	1994	1-Eicosene	C_20_H_40_	0.4 ± 0.07	0.52 ± 0.03	0.40 ± 0.02
40.	2000	2000	Eicosane	C_20_H_42_	0.47 ± 0.09	0.40 ± 0.06	0.38 ± 0.08
41.	2005	2008	1-Hexadecanol acetate	C_18_H_36_O_2_	0.15 ± 0.01	0.09 ± 0.02	0.13 ± 0.03
42.	2027	2024	Octadecanal	C_18_H_36_O	0.18 ± 0.06	0.16 ± 0.07	0.32 ± 0.04
43.	2035	2034	Geranyl linalool	C_20_H_34_O	0.15 ± 0.02	0.09 ± 0.05	0.11 ± 0.08
44.	2088	2086	1-Octadecanol	C_18_H_38_O	-	-	0.12 ± 0.03
45.	2095	2100	Henicosane	C_21_H_44_	0.34 ± 0.06	0.86 ± 0.02	0.29 ± 0.03
46.	2096	2100	1-Heneicosene	C_21_H_42_	-	0.3 ± 0.06	0.13 ± 0.03
47.	2106	2107	Linolenic acid, methyl ester	C_19_H_32_O_2_	-	0.13 ± 0.04	0.18 ± 0.09
48.	2121	2122	Phytol	C_20_H_40_O	13.56 ± 0.05	0.85 ± 0.05	7.29 ± 0.19
49.	2149	2145	Linoleic acid	C_18_H_32_O_2_	-	1.08 ± 0.10	0.81 ± 0.08
50.	2162	2164	Linoleic acid ethyl ester	C_20_H_36_O_2_	-	0.14 ± 0.01	0.26 ± 0.03
51.	2181	2185	Phytanic acid, methyl ester	C_21_H_42_O_2_	-	-	0.05 ± 0.02
52.	2194	2198	1-Docosene	C_22_H_44_	0.92 ± 0.11	0.33 ± 0.05	0.35 ± 0.08
53.	2199	2200	Docosane	C_22_H_46_	0.10 ± 0.05	0.16 ± 0.03	0.24 ± 0.05
54.	2209	2208	Octadecanol acetate	C_20_H_40_O_2_	-	-	0.08 ± 0.02
55.	2223	2219	Phytol, acetate	C_22_H_42_O_2_	0.12 ± 0.09	-	0.27 ± 0.06
56.	2301	2300	Tricosane	C_23_H_48_	0.32 ± 0.04	0.62 ± 0.01	0.87 ± 0.09
57.	2331	2333	Eicosanoic acid, methyl ester	C_21_H_42_O_2_	-	-	0.08 ± 0.02
58.	2362	2365	2-Methyl-Tricosane	C_24_H_50_	0.12 ± 0.06	0.12 ± 0.01	0.13 ± 0.05
59.	2374	2380	Eicosanoic acid	C_20_H_40_O_2_	0.11 ± 0.09	-	0.08 ± 0.01
60.	2391	2400	Tetracosane	C_24_H_50_	0.65 ± 0.06	0.50 ± 0.03	1.17 ± 0.12
61.	2391	2396	1-Tetracosene	C_24_H_48_	-	0.24 ± 0.09	0.43 ± 0.06
62.	2433	2430	Heneicosanoic acid, methyl ester	C_22_H_44_O_2_	-	-	0.06 ± 0.01
63.	2501	2500	Pentacosane	C_25_H_52_	1.32 ± 0.12	1.83 ± 0.13	5.81 ± 0.07
64.	2517	2509	Heneicosyl acetate	C_23_H_46_O_2_	0.13 ± 0.02	0.08 ± 0.02	-
65.	2536	2531	Docosanoic acid, methyl ester	C_23_H_46_O_2_	-	-	0.07 ± 0.01
66.	2599	2600	Hexacosane	C_26_H_54_	0.87 ± 0.08	1.16 ± 0.07	3.06 ± 0.05
67.	2637	2632	Tetracosanal	C_24_H_48_O	-	0.11 ± 0.03	0.13 ± 0.05
68.	2689	2684	Heptacos-1-ene	C_27_H_54_	-	-	0.06 ± 0.01
69.	2704	2700	Heptacosane	C_27_H_56_	9.41 ± 0.09	10.93 ± 0.04	23.32 ± 0.03
70.	2720	2725	Methyl tetracosanoate	C_25_H_50_O_2_	0.12 ± 0.06	-	-
71.	2790	2800	Octacosane	C_28_H_58_	2.88 ± 0.10	1.80 ± 0.09	2.05 ± 0.08
72.	2792	2797	1-Octacosene	C_28_H_56_	-	0.84 ± 0.05	2.09 ± 0.05
73.	2836	2835	Squalene	C_30_H_50_	1.28 ± 0.02	0.52 ± 0.04	1.09 ± 0.04
74.	2842	2848	Hexacosanol	C_26_H_54_O	0.06 ± 0.01	0.15 ± 0.04	-
75.	2892	2884	Nonacos-1-ene	C_29_H_58_	0.22 ± 0.04	0.13 ± 0.03	0.39 ± 0.07
76.	2906	2900	Nonacosane	C_29_H_60_	23.36 ± 0.03	24.54 ± 0.05	9.76 ± 0.02
77.	2922	2929	15-Methylnonacosane	C_30_H_62_	-	-	0.08 ± 0.02
78.	2944	2940	Hexacosanoic acid, methyl ester	C_27_H_54_O_2_	-	-	0.06 ± 0.01
79.	2963	2960	2-Methylnonacosane	C_30_H_62_	-	-	0.09 ± 0.01
80.	2999	3000	Triacontane	C_30_H_62_	0.63 ± 0.03	0.70 ± 0.11	0.44 ± 0.05
81.	3046	3040	Octacosanal	C_28_H_56_O	-	0.22 ± 0.04	0.39 ± 0.07
82.	3101	3100	Untriacontane	C_31_H_64_	4.11 ± 0.04	7.35 ± 0.09	1.42 ± 0.06
83.	3109	3110	Octacosanol	C_28_H_58_O	-	-	0.05 ± 0.01
84.	3199	3200	Dotriacontane	C_32_H_66_	-	0.08 ± 0.02	0.10 ± 0.02
85.	3245	3251	Triacontanal	C_30_H_60_O	0.31 ± 0.01	0.46 ± 0.15	0.32 ± 0.04
86.	3292	3300	Tritriacontane	C_33_H_68_	-	0.11 ± 0.08	0.08 ± 0.02
87.	3349	3351	γ-Sitosterol	C_29_H_50_O	-	0.16 ± 0.07	0.07 ± 0.01
88.	3368	3327	β-Amyrone	C_30_H_48_O	0.17 ± 0.01	0.14 ± 0.04	0.08 ± 0.01
89.	3395	3400	Tetratriacontane	C_34_H_70_	0.67 ± 0.05	0.39 ± 0.09	1.09 ± 0.04
90.	3422	3384	Lupenone	C_30_H_48_O	1.40 ± 0.07	1.16 ± 0.04	0.61 ± 0.09
91.	3451	3525	Lupeol acetate	C_32_H_52_O_2_	2.33 ± 0.11	-	-
92.	3463	3500	Lupeol	C_30_H_50_O	0.24 ± 0.03	2.33 ± 0.01	3.29 ± 0.03
Total identified compounds%					93.57	92.65	94.9

Compounds were identified according to their mass spectral data and retention indices compared to those of the NIST Mass Spectral Library and the Wiley Registry of Mass Spectral Data, 8th edition. The content (%) was calculated in triplicate using the normalization method based on the GC-MS data. The presented data are shown as the average of three replicas. (-): Unidentified in the sample. The standard deviation did not exceed 3% for any of the values. KI: Kovats index calculated on Rtx-5MS column. CL (*C. lancifolius*), CEG (*C. erectus* green leaves), and CES (*C. erectus* silver leaves).

**Table 2:** Antioxidant activity of essential oils derived from *Conocarpus* species.

Plant	DPPH Scavenging Assay	FRAP Scavenging Assay
*C. lancifolius*	470.12 ± 9.84	603.74 ± 17.54
*C. erectus* silver	349.78 ± 8.26	432.48 ± 9.56
*C. erectus* green	571.79 ± 16.31	796.38 ± 18.91

Results are reported as the mean values of three replicates and expressed as IC_50_ values (µg/mL) with ascorbic acid as the standard reference. Data are presented as mean ± standard deviation.

**Table 3:** Assessment of antimicrobial activity of three *Conocarpus* species EOs using the disc diffusion method.

Microorganism	*Conocarpus* Species			Controls	
	CL	CES	CEG	Ketoconazole	Gentamycin
*A. niger*	9	NA	NA	15	NT
*C. albicans*	13	15	12	20	NT
*S. aureus*	15	10	10	NT	24
*B. subtilis*	14	9	11	NT	26
*E. coli*	12	14	10	NT	30
*P. vulgaris*	13	12	NA	NT	25

NA: No activity, NT: Not tested. CL (*C. lancifolius)*, CEG (*C. erectus* green leaves) and CES (*C. erectus* silver leaves).

**Table 4:** Minimum inhibitory concentration (MIC) values of EOs from three *Conocarpus* species against tested microorganisms.

Microorganisms	*Conocarpus* Species EOs			Controls	
	CL	CES	CEG	Ketoconazole	Gentamycin
*A. niger*	5000	NA	NA	19.54	NT
*C. albicans*	1250	625	1250	9.77	NT
*S. aureus*	625	2500	2500	NT	4.88
*B. subtilis*	1250	5000	2500	NT	4.88
*E. coli*	1250	625	2500	NT	9.76
*P. vulgaris*	1250	1250	NA	NT	4.88

NA: No activity, NT: Not tested. CL (*C. lancifolius*), CEG (*C. erectus* green leaves), and CES (*C. erectus* silver leaves).

## Data Availability

Data are available upon request from the corresponding authors: naglaa.saad@pharma.asu.edu.eg (N.S.A.).
